# Prognostic role of platelet to lymphocyte ratio in hepatocellular carcinoma: a systematic review and meta-analysis

**DOI:** 10.18632/oncotarget.15281

**Published:** 2017-02-11

**Authors:** Yongzhao Zhao, Guangyan Si, Fengshang Zhu, Jialiang Hui, Shangli Cai, Chenshen Huang, Sijin Cheng, Abdel Hamid Fathy, Yi Xiang, Jing Li

**Affiliations:** ^1^ School of Medicine, Tongji University, Shanghai, China; ^2^ Department of Interventional Radiology, Affiliated Traditional Chinese Medicine Hospital, Southwest Medical University, Luzhou, China; ^3^ Department of Gastroenterology, Tongji Hospital, Tongji University, Shanghai, China; ^4^ Department of General Surgery, Nanfang Hospital, Southern Medical University, Guangzhou, China; ^5^ Mental Health Institute of the Second Xiangya Hospital, National Technology Institute of Psychiatry, Key Laboratory of Psychiatry and Mental Health of Hunan Province, Central South University, Hunan, China

**Keywords:** platelet to lymphocyte ratio, hepatocellular carcinoma, prognostic, overall survival

## Abstract

**Background and Aims:**

Several studies were conducted to explore the prognostic significance of platelet to lymphocyte ratio (PLR) in hepatocellular carcinoma (HCC), however, contradictory results across most reports were documented. To this end, we present a systematic review that aims to summarize the prognostic significance of PLR in patients with HCC.

**Results:**

A total of 10 studies involving a total of 2,315 patients were identified. The Newcastle-Ottawa Quality Assessment Scale (NOS) of each included study was greater than or equal to 5. The results indicated that high PLR was significantly associated with a worse OS when compared to the low PLR (HR = 1.60, 95% CI = 1.23−2.08, *p* = 0.0005; I^2^ = 88%, *p* < 0.00001). Similar results were detected in the subgroup analysis of the analysis model, cut-off value, ethnicity, sample size and therapy. However, no obvious correlation between the PLR and DFS/RFS in patients with HCC was observed (HR = 1.21, 95% CI = 0.87−1.67, *p* = 0.26; I^2^ = 61%, *p* = 0.07).

**Materials and Methods:**

A complete literature search in the PubMed, Cochrane Library and Embase database was performed. Retrospective and prospective studies focusing on the role of PLR on the prognosis in HCC were all deemed as “suitable” for our scope. The endpoints determined were: the overall survival (OS), disease-free survival (DFS), recurrence-free survival (RFS) and the progress free survival (PFS).

**Conclusions:**

The study revealed that high PLR is an unfavorable predictor of OS in patients with HCC, and high PLR is a promising prognostic biomarker for HCC, especially for patients in Asia.

## INTRODUCTION

HCC is a principal health problem all over the world. It is estimated that there were 782,500 new liver cancer cases and 745,500 deaths occurred worldwide during 2012 [1]. Of which 466,100 new liver cases and 422,100 liver related deaths occurred in China. Hepatocellular carcinomas (HCC) dominates the majority of liver cancer, accounting around 85% of all primary liver neoplastic diseases [2]. Liver cirrhosis is the cornerstone of HCC in 80% of the cases [3]. Numerous factors have been proved to be associated with the occurrence of HCC, such as HBV infection, obesity, and so on [4]. As for patients diagnosed at early stage, the mainstream treatments are tumor resection, thermal ablation (TA) and liver transplantation (LT), with 5-year survival rates of around 50%. Yet, a number of patients were at advanced stage when diagnosed, who have to receive the transarterial chemoembolization (TACE) or systematical chemotherapy and have very poor clinical outcomes [5]. Therefore, identification of the finest diagnostic biomarkers for better prognosis of HCC, is being recognized as a promising direction to improve the survival rate of patients with HCC. Various studies have confirmed that inflammation is a vital component of the growth, invasion, and metastasis of tumors [6–8]. Systemic inflammatory response (SIR) has been proved to be associated with prognosis of various tumors [9–14]. Recently, several studies reported that the platelet to lymphocyte ratio (PLR) was correlated with prognosis of multiple kinds of tumors, such as lung cancer, colorectal cancer, esophageal cancer and so on [15–18]. As for HCC, the study conducted by *Fan et al*. supported that high PLR was an unfavorable factor on the prognosis and associated with shorter OS when compared to the low PLR [19]. Whereas,, *Xue et al*. explored the prognostic role of PLR in HCC, and declared that patients with high PLR had worse OS when compared with patients with low PLR [20]. However, no evident association between the PLR and OS in HCC was distinguished in the study conducted by *Kabir et al*. [21]. Therefore, controversy does exist on the prognostic role of PLR in HCC. To this extent, the aim of this systematic review and meta-analysis is to explore the prognostic role of PLR in HCC.

## RESULTS

### Literature search

**Figure 1:**
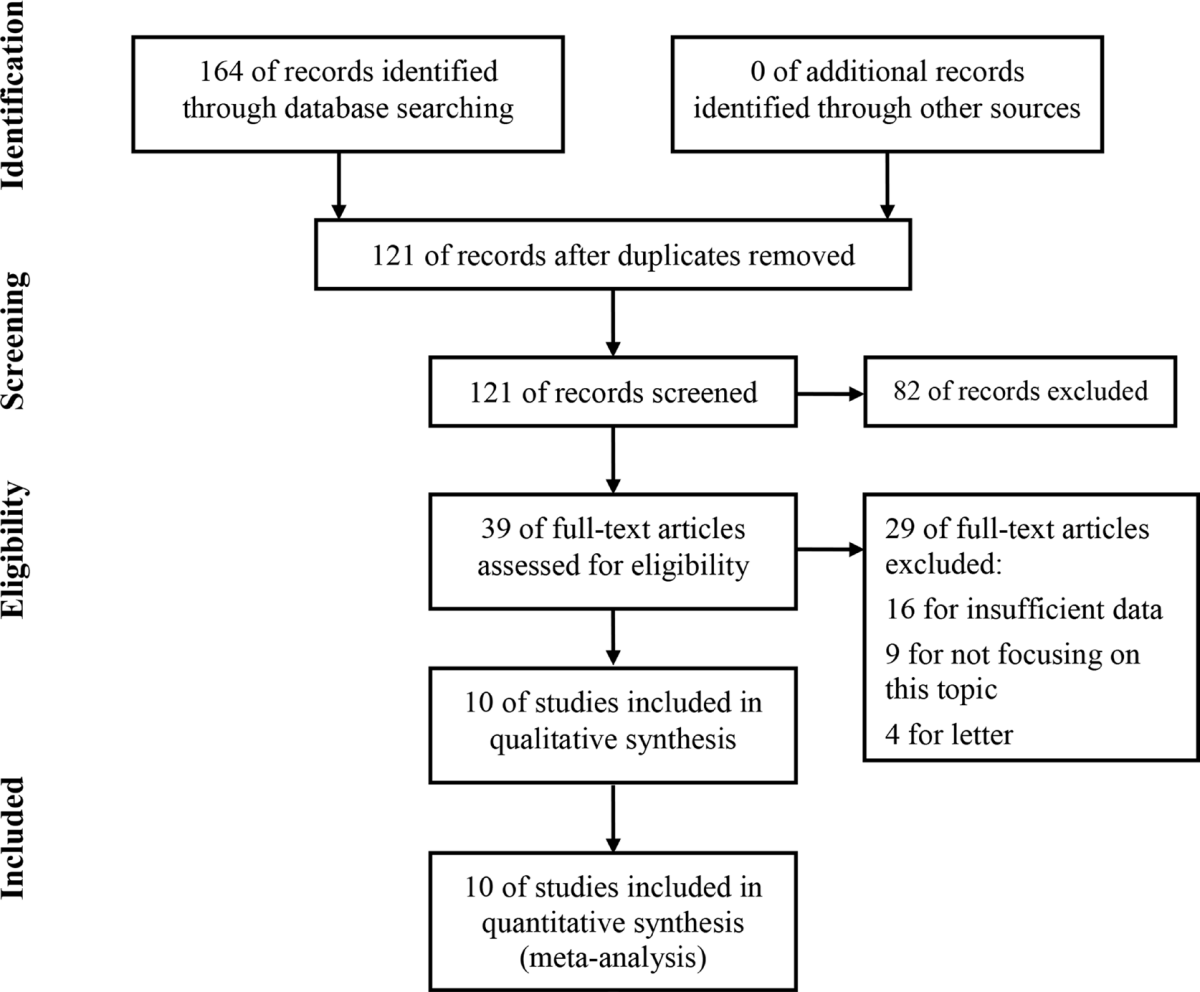
Flow diagram of study selection process

As shown in Figure 1, a total of 164 papers were identified of which 43 duplicative papers were excluded. As for the remaining 121 papers, 82 were excluded by scanning either the titles or abstracts. For the 39 remaining potentially related studies, the full-text was carefully read. 16 were excluded for insufficient datum to assess the HR of prognosis outcomes, and 7 were excluded for not focusing on this topic, and 1 was excluded because the included patients were all covered by the study conducted by Kaprio et al., which published in the *BMC cancer*. At last, 10 studies involved 2,315 patients were eligible for this meta-analysis [19, 20, 22–29].

### Characteristics of included studies

As listed in Table 1, the ten included studies contained 2,315 patients. As for assessment of included studies, the NOS of six included studies was 7 and four included studies was 5 (Table 3). and the Global score of each included study was more than 60.00% (Supplementary Table 1). The median age of patients varies from 47 to 67 year-old. Three studies focused on the role of PLR on the prognosis of the surgery [23, 24, 28], three studies paid attention to the TACE [19, 20, 29], and one study contained patients receiving TA[26]. Besides, two studies involved patients treated with various therapies [22, 27] and one study did not report the treatment of patients [25]. In addition, the sample size was different, varying from 80 patients to 434 patients. In term of ethnicity, nine studies focused on the Asian [19, 20, 22–26, 28, 29] and one study focused on Caucasians [27]. As for survival analysis, eight studies reported the OS [19, 20, 22–25, 27, 29], two studies covered the RFS [23, 26] and one study reported the DFS [28]. Furthermore, all the included studies reported the value of cut-off, varying from 87.87 to 300.

**Table 1:** Characteristics of the included studies

Study	Year	Country	Ethnicity	Patients (*n*)	Male (%)	Age (years)	Treatment	Outcome	Cut-off	Analysis
Pinato et al [28]	2012	UK	Caucasian	112	80.0	65 (20–83)	VT	OS	300	U
Sun et al [29]	2014	China	Asian	80	95.0	47 (29–72)	Surgery	DFS	151.8	M
Li et al [26]	2014	China	Asian	243	86.8	57 (19–86)	NR	OS	111.23	M
Fan et al [19]	2015	China	Asian	132	65.9	49 (23–75)	TACE	OS	137	M
Li et al [27]	2015	China	Asian	414	83.1	59.5 (28–82)	TA	RFS	87.87	M
Xue et al [20]	2015	China	Asian	291	88.7	53	TACE	OS	150	M
Aino et al [23]	2016	Japan	Asian	434	83.6	67 (15–92)	VT	OS	111	U
Goh et al [24]	2016	Singapore	Asian	166	85.5	66 (21–85)	Surgery	RFS,OS	290	U
Ji et al [25]	2016	China	Asian	321	88.8	51 (21–79)	Surgery	OS	115	M
Tian et al [30]	2016	China	Asian	122	87.7	56 (26–77)	TACE	OS	96.13	M

Abbreviations: VT, various therapies, including TA, surgery, chemotherapy and so on; NR, not reported; TACE, transarterial chemoembolization; TA, thermal ablation; OS, overall survival; DFS, disease-free survival; RFS, recurrence-free survival; U, univariate; M, multivariate; NOS, Newcastle-Ottawa Scale.

### Meta-analysis of OS

**Figure 2:**
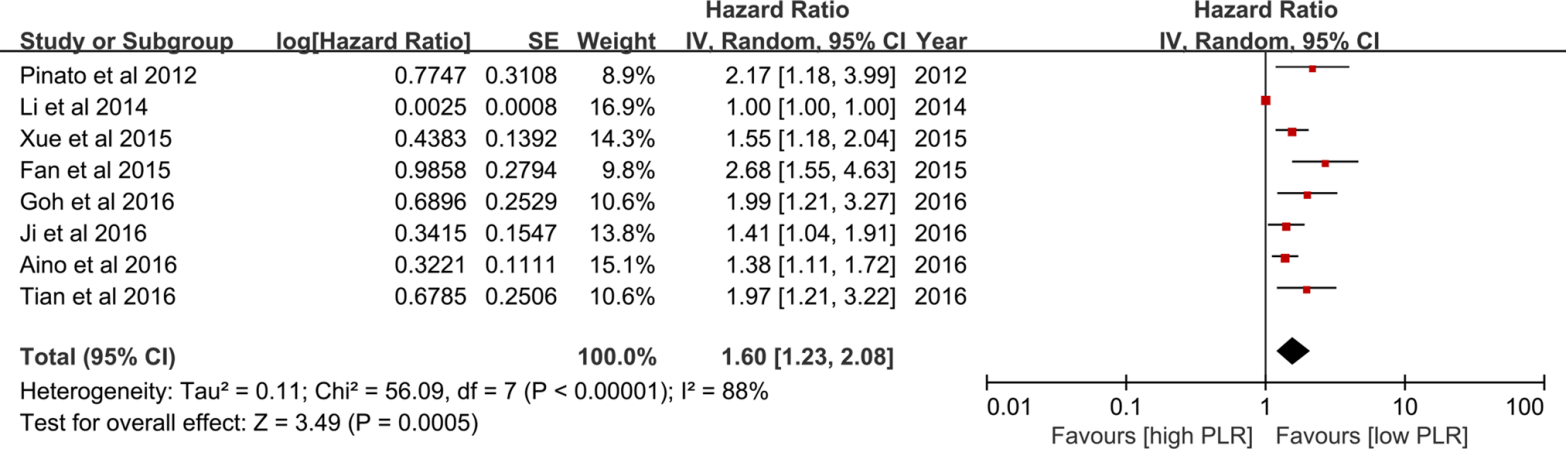
Meta-analysis of overall survival

Eight studies involving 1,821 patients were included in the meta-analysis of OS. As showed in Figure 2, in view of the significant heterogeneity (I^2^ = 88%, *p* < 0.00001), the random-effect model was used. A significant correlation between the PLR and OS was observed (HR = 1.60, 95% CI = 1.23–2.08, *p* = 0.0005), and the result revealed that high PLR predicted worse OS when compared with the low PLR.

As listed in Table 2, the subgroup analyses were carried out to investigate the sources of heterogeneity. As for the included studies assessed by multivariate analysis, the result showed that high PLR was significantly associated with shorter OS when compared with the low PLR, with obvious heterogeneity (HR = 1.54, 95% CI = 1.09–2.18, *p* = 0.01; I^2^ = 88%, *p* < 0.00001). Similarly, significant association between the PLR and OS was identified for the included studies assessed by univariate analysis, when using the fixed-effect model (HR = 1.52, 95% CI = 1.26–1.84, *p* < 0.0001; I^2^ = 38%, *p* = 0.20). As for the subgroup analysis of ethnicity, the results indicated that high PLR appeared to be a stronger predictor of risk when compared to low PLR in Asians, using the random-effect model (HR = 1.55, 95% CI = 1.18–2.03, *p* = 0.001; I^2^ = 88%, *p* < 0.00001). And the result also presented that high PLR was an unfavorable factor for prognosis in Caucasians (HR = 2.17, 95% CI = 1.18–3.99, *p* = 0.01). Subgroup analysis stratified by PLR cut-off value showed that the high PLR was a risk factor both when cut-off value < 150 (HR = 1.48, 95% CI = 1.09–2.02, *p* = 0.01; I^2^ = 88%, *p* < 0.00001) and cut-off value ≥ 150 (HR=1.71, 95% CI = 1.37–2.13, *p* < 0.00001; I^2^ = 0%, *p* = 0.48). In terms of sample size, the result showed that PLR was obviously associated with OS and high PLR was an unfavorable factor for prognosis in HCC (HR = 1.37, 95% CI = 1.06–1.78, *p* = 0.02; I^2^ = 87%, *p* < 0.00001) when sample size ≥ 150. And similar result was detected between the patients with high PLR and patients with low PLR when sample size < 150, using fixed-effect model (HR = 2.24, 95% CI = 1.63–3.06, *p* < 0.00001; I^2^ = 0%, *p* = 0.71). The subgroup analysis also revealed that the statistical correlation between the PLR and OS was observed when the following treatment was TACE, using fixed-effect model (HR = 1.77, 95% CI = 1.43–2.21, *p* < 0.00001; I^2^ = 39%, *p* = 0.19). Similar results were observed in term of surgery, using fixed-effect model (HR = 1.55, 95% CI = 1.19–2.00, *p* = 0.0009; I^2^ = 27%, *p* = 0.24). Other than that, sensitivity analysis indicated that the combined HRs of OS did not significantly alter when excluding any included study (Supplementary Figure 1). And the funnel plot was conducted to assess the publication bias (Supplementary Figure 2).

**Table 2:** The main results of subgroup analysis

Terms	Included studies	HR 95% CI	*p*	I^2^	*p* value for heterogeneity
**Analysis**					
univariate	3	1.52 [1.26, 1.84]	< 0.0001‡	38%	0.20
multivariate	5	1.54 [1.09, 2.18]	0.01‡	88%	< 0.00001
**Cut-off value**					
< 150	5	1.48 [1.09, 2.02]	0.01‡	88%	< 0.00001
≥ 150	3	1.71 [1.37, 2.13]	< 0.00001‡	0%	0.48
**Ethnicity**					
Asian	7	1.55 [1.18, 2.03]	0.001‡	88%	< 0.00001
Caucasian	1	2.17 [1.18, 3.99]	0.01‡	NA	NA
**Sample Size**					
< 150	3	2.24 [1.63, 3.06]	< 0.00001‡	0%	0.71
≥ 150	5	1.37 [1.06, 1.78]	0.02‡	87%	< 0.00001
**Therapy**					
TACE	3	1.77 [1.43, 2.21]	< 0.00001‡	39%	0.19
Surgery	2	1.55 [1.19, 2.00]	0.0009‡	27%	0.24

Abbreviations: TACE, transarterial chemoembolization; NA, not applicable; ‡, p < 0.05 and the difference was significant.

**Table 3:** Assessment of study quality

Study	Quality indicators from the Newcastle-Ottawa scale									Score
	Selection				Comparable		Outcome assessment			
	1	2	3	4	5	6	7	8	9	
Pinato et al[28]	*	*	*				*	*		5
Sun et al[29]	*	*	*		*	*	*	*		7
Li et al [26]	*	*	*		*	*	*	*		7
Fan et al [19]	*	*	*		*	*	*	*		7
Li et al [27]	*	*	*			*	*			5
Xue et al [20]	*	*	*		*	*	*	*		7
Aino et al [23]	*	*	*				*	*		5
Goh et al [24]	*	*	*		*	*	*	*		7
Ji et al [25]	*	*	*				*	*		5
Tian et al [30]	*	*	*		*	*	*	*		7

*For cohort studies, 1 indicates exposed cohort truly representative; 2, non-exposed cohort drawn from the same community; 3, ascertainment of exposure; 4, outcome of interest not present at start; 5, cohorts comparable on basis of age; 6, cohorts comparable on other factor(s); 7, quality of outcome assessment; 8, follow-up long enough for outcomes to occur; and 9, complete accounting for cohorts.

### Meta-analysis of RFS/DFS

**Figure 3:**
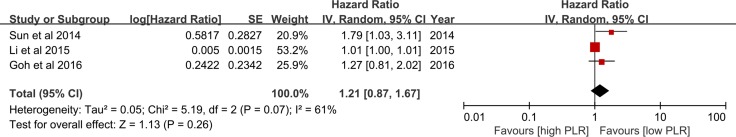
Meta-analysis of recurrence-free survival / disease free survival

Two studies reporting the RFS and one study covering the DFS of patients with HCC were all included into the meta-analysis. As shown in Figure 3, no evident relationship was observed between the PLR and RFS/DFS (HR = 1.21, 95% CI = 0.87–1.67, *p* = 0.26; I^2^ = 61%, *p* = 0.07), with significant heterogeneity. Besides, there was no decisive effect according to the influence analysis (Supplementary Figure 3), and no significant bias among all included studies was detected by funnel plot (Supplementary Figure 4).

## DISCUSSION

Inflammation has been proved to play a vital role in tumor growth, invasion and metastasis [30]. Many inflammatory indicators that were explored to predict the prognosis in various cancers, such as neutrophil-to-lymphocyte ratio (NLR), PLR, C-reactive protein and so on, have greatly contributed to our understanding in pathogenesis of tumorous diseases [31–36]. As for PLR, it had been extensively researched in various tumors and considered as a promising prognostic factor [16, 17, 37–39]. Even though many studies focusing on the prognostic role of PLR in HCC were carried out [19–29], most findings were contradictive, which drives us to study the discrepancies and draw a proper conclusion.

In our study, the results showed that PLR was obviously associated with OS, and high PLR predicted shorter OS when compared with the low PLR in HCC. The correlation between the high PLR and worse OS remains significant in the subgroup analysis of the analysis model, sample size and value of cut-off, which made the conclusion more convincing.

Our study revealed that the significant correlation between the PLR and OS was observed both in the Asian and Caucasian population. Besides, *Kinoshita et al* revealed that high PLR was related with worse OS in patients in Japan (*p* < 0.0001), but was excluded from the meta-analysis for only reporting the relevant *p* values [40]. Similar result was detected in *Ji et al*. study (*p* = 0.005) [41] and *Ni et al*. study both conducted in China (*p* = 0.01) [42], in which OS was assessed by univariate analysis. And *Yang et al*. study excluded from the meta-analysis for not reporting HR also covered that elevated PLR was related to shorter OS (*p* = 0.02) [43]. However, no evident association between the PLR and OS was found in the study conducted by *Kabir et al*. in Singapore (*p* = 0.341) [21], and a similar result was detected in *Peng et al*. study in China (*p* = 0.856) [44], however, they were both excluded from the meta-analysis because they only reported the relevant *p* values without HR. Therefore, more prospective cohort studies should be carried out to explore the prognostic role of PLR in HCC in Asian. As for the studies focusing on the Caucasian patients, only one study conducted by *Pinato et al*. was included into the meta-analysis and reported the association between the PLR and OS [27]. And *Pinato et al*. yielded the conclusion that high PLR predicted worse OS when compared to the low PLR. It must noticed that only 122 patients were included into the study and their treatments were various. Besides, the value of cut-off was 300, which was the largest among all the included studies. Moreover, the prognostic role of PLR in the *Pinato et al*. study was assessed by univariate analysis not multivariate analysis [27]. Therefore, the conclusion of relationship between the PLR and OS in Caucasian should be yielded with caution, and more studies focusing on the prognostic role of PLR in HCC should be carried out on Caucasians.

Our study reported that high PLR predicted a worse OS in patients undergoing surgery with HCC. Similar results were observed in *Shen et al*. study (*p* = 0.007) [45] and *Ni et al*. study (*p* = 0.01) [42]. However, different result was detected in *Peng et al*. study, which revealed that no significant difference between the PLR and OS was found. But all the included patients in *Peng et al*. study were with HBV-related small HCC, which might have caused the difference between its outcome and the result of our study [44]. Additionally, our study indicated that high PLR was an unfavorable factor for patients receiving TACE with HCC. Though, *Zhou et al*. study, which involved 224 patients undergoing TACE, reported that no evident relationship was found between the PLR and OS in HCC, however, *Zhou et al*. study was excluded from the meta-analysis for only reporting the relevant *p* values [46], and the primary data was difficult to obtain though we have tried to contact the authors. Therefore, more studies focusing on the prognostic role of PLR in HCC resection should be actualized. As for patients receiving the treatment of liver transplantation, *Xia et al*. reported high PLR was associated with worse OS (*p* = 0.012) [47], and similar result was presented by *Yang et al*. [48]. But both of them were not included into our study because of the insufficient datum.

In our study, no obvious relationship between the PLR and RFS/DFS was observed. More to the point, *Parisi et al*. [49] and *Kabir et al*. [21] both covered that PLR was not evidently associated with the RFS of HCC. However, *Ji et al*. declared the low PLR was a favorable prognostic factor in term of DFS in HCC [41]. Hence, in view of the dispute, more effort should be made to research the prognostic role of PLR on RFS/DFS in HCC.

The highlighted strength of the meta-analysis is as follows: Primarily although a recent meta-analysis has been conducted to explore the prognostic role of PLR in HCC, however, there are several major differences in our study compared to theirs [50]. First, we have enrolled three studies (Pinato et al. [28], Sun et al. [29], Aino et al. [23]), which they haven't. Second, they enrolled a very inappropriate study conducted by Peng et al., which has been carried out to determine the significance of the decreased range between pre-operation and post-operation PLR values in prognosis of patients with HCC. All the residual studies have been focused on the clinical significance of pre-operation PLR values. Therefore, our study was necessary to update the previous meta-analysis to explore the prognostic role of PLR in HCC. Secondly, ten studies involving 2,315 patients were finally included, thus, the results were large enough to be valid. Above and beyond that, a comprehensive subgroup analyses was carried out, such as PLR cut-off, ethnicity, therapies and sample size.

In spite though, it is also to be noted that several limitations of our study should be carefully considered. Firstly, all the included studies were retrospective, which might increase possibility of bias into our study. Then, all data was obtained from the published articles, leading to our inability assess each individual's data, such as dose of drug, stage of HCC, and so on. Thirdly, the heterogeneity remains significant though subgroup analysis when calculated. And random-effect model was applied, which might reduce the accuracy of the results. Fourthly, the therapy and the value of cut-off varied a lot, which might bring down reliability. At last, moderate publication bias existed because the researchers tended to report the complete datum of positive results.

In conclusion, though no significant correlation between the PLR and RFS/DFS was observed, the study revealed that high PLR is an unfavorable predictor of OS in patients with HCC, and high PLR is a promising prognostic biomarker for HCC, especially for patients in Asian countries.

## MATERIALS AND METHODS

### Literature search strategy

We performed a complete computer-based search of the PubMed, Embase and the Cochrane Library databases for clinical trials in original articles up to the date of September 29, 2016. The search strategy was conducted according to a combination of the following terms: “(((((((liver neoplasm) OR liver cancer) OR cancer of Liver) OR hepatocellular Cancer) OR hepatic Cancers) OR hepatocellular carcinoma)) AND ((((PLR) OR platelet-to-lymphocyte ratio) OR platelet lymphocyte ratio) OR platelet-lymphocyte ratio)”. The irrelevant articles were directly excluded by scanning the titles or abstracts. We also examined reference lists of selected field for each original article that may fulfill our eligibility requirements in order to avoid missing relevant studies. The remaining articles were then reviewed comprehensively by reading the full text.

### Inclusion criteria

Studies meeting all the following criteria were included: 1) retrospective or prospective studies; 2) paid attention to the role of PLR on the prognosis in HCC; 3) providing enough data to get the hazard ratio (HR) for prognosis outcomes, along with their 95% confidence intervals (CIs) or *p* values; 4) published in English.

### Exclusion criteria

The exclusion criteria were as follows: 1) neither the retrospective nor prospective studies; 2) studies without sufficient data to pool the HR; 3) studies not focusing on the role of the PLR on the prognosis in HCC; 4) not published in English.

### Data abstraction and quality assessment

Two investigators (Zhu F and Zhao Y) independently fully reviewed all the manuscripts. The following datum were abstracted: first name of the author, publication year, country of the study, ethnicity of patients in the study, sample size, cut-off value of PLR, treatment, survival analysis. The HRs of disease-free survival (DFS), recurrence-free survival (RFS), progress free survival (PFS) or overall survival (OS) obtained directly or indirectly from published articles were integrated in the meta-analysis according to the study conducted by *Tierney et al*. [51]. The HR assessed by multivariate analysis was abstracted when the multivariate analysis and univariate analysis were both provided. The Newcastle-Ottawa Quality Assessment Scale (NOS) was applied to assess the quality of each included study. And NOS scores ≥ 6 are considered to show high-quality studies. Any discrepancy was discussed with the third investigator (Li J).

### Statistical analysis

Pooled analyses were carried out by Review Manager Version 5.3 software. The prognosis outcomes were explored using the HR and the corresponding 95% CI. The prognosis outcomes mainly contained the DFS, RFS or OS. The heterogeneity was assessed across all studies by Cochran's *Q* test and Higgins I^2^. The heterogeneity was significant when *p* < 0.05 and/or I^2^ > 50%, and the random-effect model was used; if not, the fixed-effects model was applied. In addition, the funnel plot was conducted to evaluate bias by Review Manager Version 5.3 software. The sensitivity analysis was conducted by Stata 12.0 to assess the robustness of the results. All the *p* was two-side and *p* < 0.05 was considered statistically significant.

## SUPPLEMENTARY FIGURES AND TABLE



## Abbreviations

PLR: platelet to lymphocyte ratio
HCC: hepatocellular carcinoma
OS: overall survival
DFS: disease-free survival
RFS: recurrence-free survival
PFS: progress free survival
TA: thermal ablation
LT: liver transplantation
TACE: transarterial chemoembolization
SIR: systemic inflammatory response
HR: hazard ratio
