# Supplementary material for: Prognostic role of platelet to lymphocyte ratio in hepatocellular carcinoma: a systematic review and meta-analysis
Source: Oncotarget. 2017 Feb 11;8(14):22854–62. doi: 10.18632/oncotarget.15281 (PMC5410268; doi:10.18632/oncotarget.15281)
Supplement: Supplementary file 1 [file oncotarget-08-22854-s001.pdf]

Prognostic role of platelet to lymphocyte ratio in hepatocellular carcinoma: a systematic review and meta-analysis

Supplementary Materials

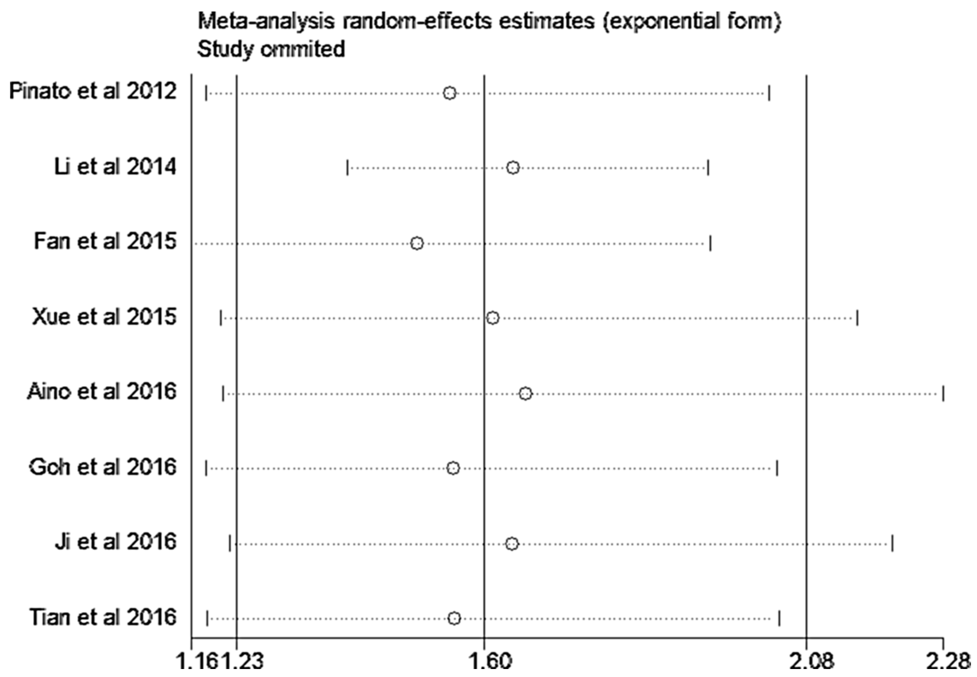

Supplementary Figure 1: Influence analysis of overall survival.

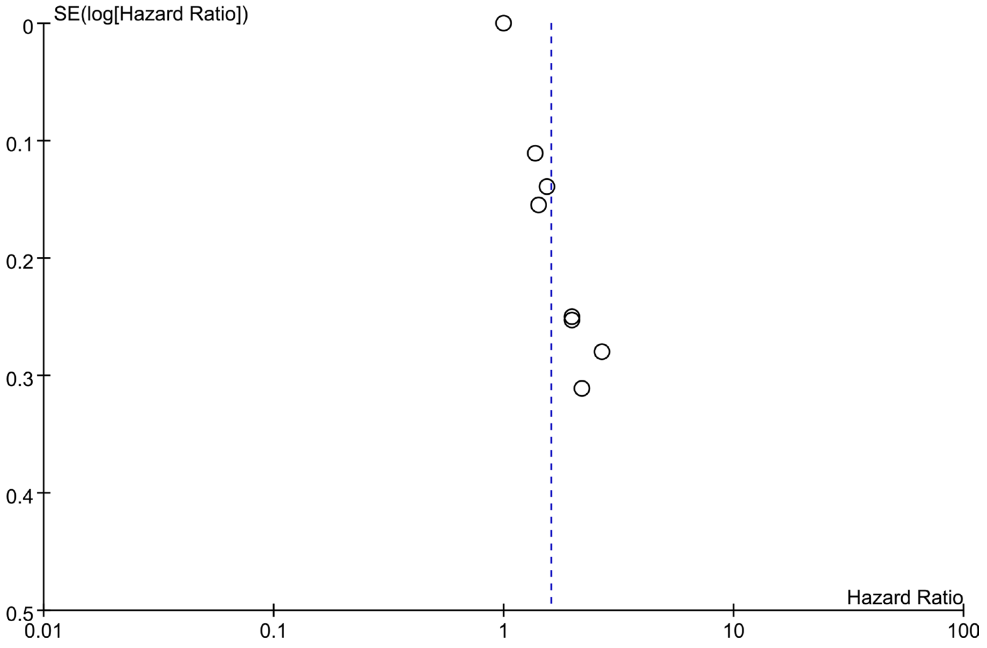

Supplementary Figure 2: Funnel plot of overall survival.

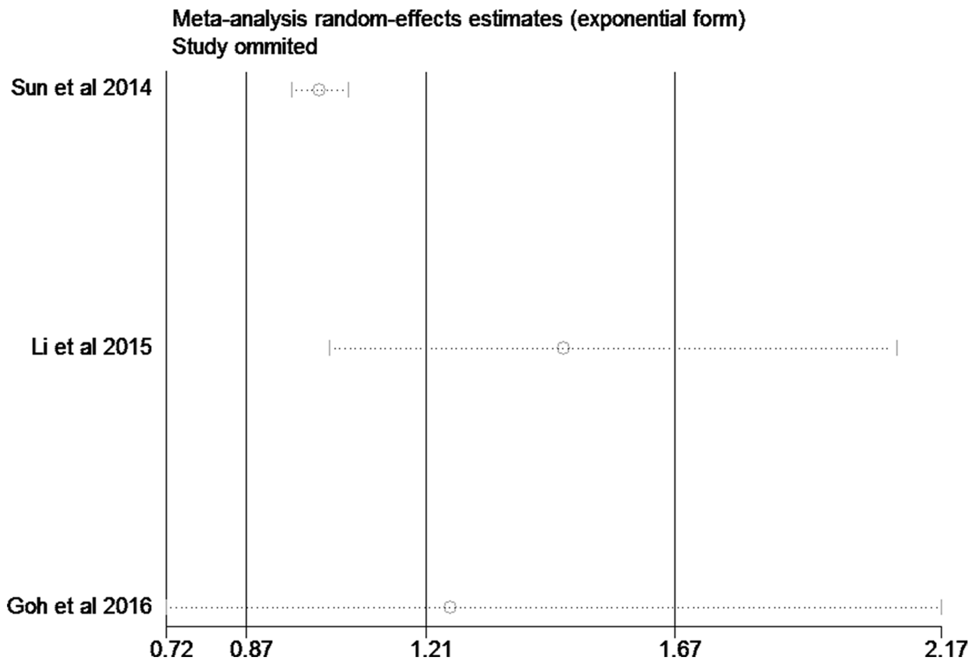

Supplementary Figure 3: Influence analysis of recurrence-free survival/disease free survival.

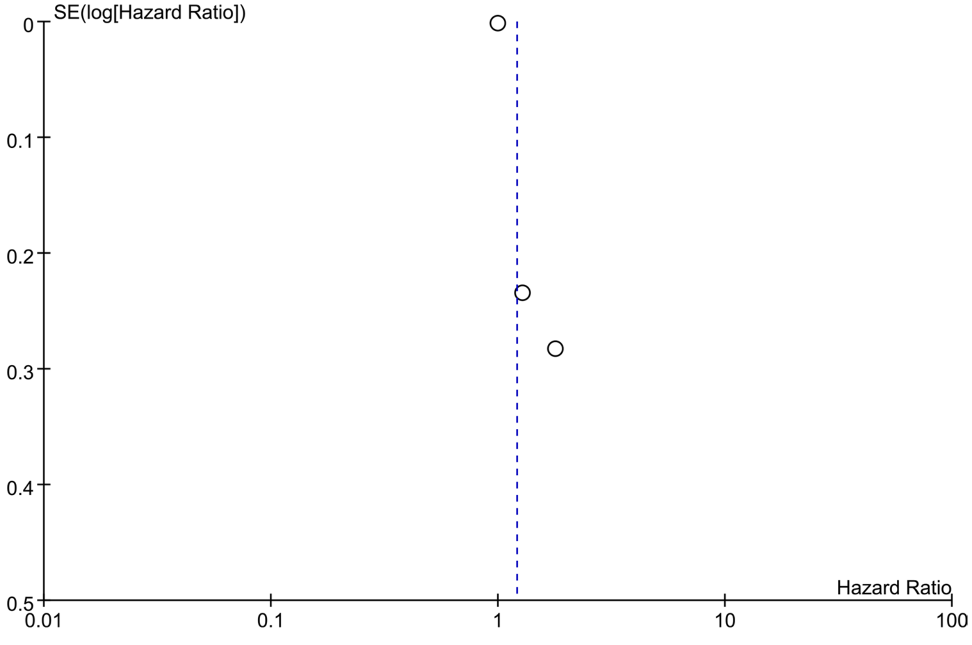

Supplementary Figure 4: Funnel plot of recurrence-free survival/disease free survival.

Supplementary Table 1: Quality assessments of the included studies in the meta-analysis

Author	Global score (%)	Scientific design (/10)	Laboratory methodology(/14)	Generalizability (/12)	Results analysis (/8)
Pinato et al. [28]	65.91	7	6	11	5
Sun et al. [29]	70.45	8	6	10	7
Li et al. [26]	70.45	8	4	11	8
Fan et al. [19]	72.73	7	6	12	7
Li et al. [27]	61.36	7	6	10	4
Xue et al. [20]	75.00	9	6	10	8
Aino et al. [23]	72.73	9	4	12	7
Goh et al. [24]	65.91	8	6	8	7
Ji et al. [25]	70.45	9	6	10	6
Tian et al. [30]	72.73	8	6	10	8
